# Head dimensions in children with and without cerebral palsy in Kano

**DOI:** 10.4314/ahs.v25i2.31

**Published:** 2025-06

**Authors:** Abdulsalam Hassana, Tela Idris Abdu

**Affiliations:** Bayero University College of Health Sciences, Anatomy

**Keywords:** Head dimensions, no cerebral palsy, Kano

## Abstract

**Background:**

Cerebral palsy (CP) is a chronic motor function disorder which occurs in children.

**Aim:**

To evaluate the head dimensions in children who have CP and those without in Kano.

**Methodology:**

A cross-sectional study of 110 normal and 70 children who have CP. The age, sex, head breadths (HB), head circumferences (HC), and head lengths (HL) were obtained. The data were analyzed in Minitab 17.0 and expressed as mean ± Standard Deviation (SD). Student's -t-test and Pearson's correlations were used to determine the differences and relationships between variables. P-values <0.05 was considered significant.

**Results:**

Children without CP had significantly larger head dimensions (p < 0.05) compared to those with CP. No sexual dimorphism (p > 0.05) was observed in head dimensions for children with or without CP. In children with CP, age showed significant correlations (p < 0.05) with head dimensions, with the strongest correlation found for head circumference (p < 0.001). Head length and head breadth were significantly correlated (p < 0.001) in both groups, with a stronger correlation in children with CP.

**Conclusion:**

This study concludes that children with cerebral palsy (CP) have smaller head dimensions compared to non-CP children, however, no observed sexual dimorphism observed among the two groups.

## Introduction

Cerebral palsy (CP) is a chronic motor function disorder which occurs predominantly in children as a result of nonprogressive insult to the immature brain^1^. It frequently associates with disorders of sensation, perception, cognition, communication, behaviour, epilepsy and secondary musculoskeletal problems^2^. These co-morbidities affect the overall health and quality of life in children by determining their participation in most aspects of life including schooling^3^. According to the Centre for Disease Control and Prevention (CDC), the incidence of CP varies across different geographical zones and had an average prevalence of 1-4 children for every 1,000 live births^4^. In Nigeria, as in many developing countries, the exact incidence of cerebral palsy (CP) remains unknown. A previous study by Animashaun^5^ in Lagos estimated the prevalence rate at 10 per 1000 children. Meanwhile, a community-based survey in Enugu reported a prevalence of 5 per 1000 among children under 16 years old^6^. The prevalence was also reported higher in boys than girls (male/female ratio 1.4:1)^7^. It was estimated that the majority of children with disabilities in Africa do not go to school at all^8^, and of the 72 million primary-aged children worldwide that are out of school, one-third have disabilities^9^. Several anthropometric and cephalometric parameters of normal and cerebral CP children have been studied with variable outcomes. For instance, studies in anthropometric measurements in body weight, height, head circumference, mid-arm circumference (MAC), waist circumference (WC), skin fold thickness, and subscapular skinfold thickness between the cerebral CP and control groups have reported variable findings.^10^ Also, the body composition of the pre-pubertal children with cerebral palsy was reported to be significantly lower than that of the normally developed pre-pubertal children^11^. The marked decrease in anthropometric measurements in CP patients as regards body weight, height, and head circumference may be attributed to nutritional as well as non-nutritional factors^12^-^13^. The cephalometric studies on the angular and linear measurements made on radiographs reported that a group of cerebral CP subjects presented skeletal patterns well within normal limits^14^. Also, a cephalometric study of cerebral CP children found no differences in facial height but an increase in facial depth as compared with average values for normal children^15^. They also found that composite cephalometric tracings of the cerebral palsy group and a group of normal children superimposed almost identically, suggesting that there was little difference in the skeletal form of the head between the two groups^15^. Despite numerous published anthropometric pieces of research on children who have CP, there is paucity of published literature compared the head dimensions of CP and normal children in Nigeria. Therefore, the present study aimed to evaluate the head dimensions in children with and without cerebral palsy children in Kano. The study may provide baseline data for easy diagnosis of the basic anthropometric head dimensions of normal cerebral CP patients in Kano, Nigeria.

## Subjects and Methods

### Study Design and Setting

This was a prospective case control study conducted at the Outpatient Department of Hasiya Bayero Paediatrics Hospital (HBPH) and Alkhair Islamic Academy Kano, Nigeria in June 2021. Hasiya Bayero Paediatric Hospital Kano is a privately established paediatric hospital in Kano city that provides adequate care to infants who are born prematurely and delivers excellent healthcare to the sick ones. It is the largest non-government-owned hospital in northern Nigeria and has the highest patient patronage in the region. In addition to essential paediatric services, it has both domestic and international research collaborations with universities and other research institutes. It provides outpatient clinic services weekly, every Monday between 8:30 am-10:30 am. Alkhair Islamic Academy Kano is a private school located at 74 Ibrahim Khalil St, Kano, Ungogo, Nigeria. It operates every day weekly from 7:00 am to 6:00 pm except for the weekend when the school operates between 1:00 pm and 6:00 pm.

### Ethical Consideration

The study was approved by the local Ethics Committee (NHREC/17/03/2018). Parents and guardians of participating children were approached for consent to participate in this study. The study was discussed with them and written informed consent was taken by the investigators.

### Subjects

The study included a total of 170 subjects, comprising 70 outpatient children with cerebral palsy (CP) who were receiving care at Hasiya Bayero Paediatrics Hospital (HBPH), and 100 apparently healthy children without CP from schooling at Alkhair Islamic Academy, were used as the control for the study.

### Inclusion and Exclusion Criteria

#### Children with CP

Children aged 2 to 12 years diagnosed with cerebral palsy, attending the Paediatric Neurology Outpatient Clinic, with or without epilepsy and on mono- or no antiepileptic drug therapy were included in the study. However, children with chronic systemic illnesses unrelated to CP (e.g., cardiac, renal, or pulmonary disorders, hemoglobinopathies), acute illnesses that could affect nutritional status (e.g., severe gastroenteritis within two weeks), those hospitalized during the study, those on medications known to influence growth (e.g., steroids, quinolones, methylphenidate), or with congenital malformations (e.g., cleft lip/palate) that could affect food intake were excluded.

#### Children without CP

Apparently healthy children with no history of cerebral palsy (CP) or chronic illnesses (such as cardiac conditions, chronic lung disease, or hemoglobinopathy) and no other diseases known to affect growth were randomly selected from Alkhair Islamic Academy, Kano, to serve as the control group.

#### Anthropometry

All measurements were done following the methods described by Eboh and Igbigbi.16 The head circumference was measured around the head in centimetres as the occipo-frontal circumference at maximum horizontal dimension, using an inelastic tailor's tape. The head length was measured in centimetres as the linear distance between the glabella and the opisthocranion, using the spreading calliper (ORION, Japan). The head breadth was also measured in centimetres as the biparietal diameter at the most lateral points, also with the spreading calliper.

### Data collected

The data obtained from the children include the age, sex, head circumference, head breadth and head lengths of the normal and cerebral palsy patients.

### Statistical Analyses

The data pooled were analyzed with Minitab 17.0. The age, head circumferences, head breadths and head length were expressed as mean ± Standard Deviation (SD), minimum and maximum to determine the baseline. Student-t-test was used to determine the sexual dimorphisms between variables of the participants. The relationships between the head dimensions were determined using Pearson's correlation. P < 0.05 was considered statistically significant.

## Results

Table 1 compares the age and head dimensions between children who have CP and the comparison group without CP. The age of the participants did not differ significantly (p = 0.543) however, the mean head length, head breadth and head circumference were significantly higher in the comparison group (p < 0.001).

**Table 1:** Comparison of Age and Head Dimensions Between Children with and Without Cerebral Palsy

Variables	Mean ± SD		t-value	*p* -value

	Children without Cerebral Palsy (n=110)	Children with Cerebral Palsy (n=70)		
Age (year)	05.34 ± 2.35	05.05 ± 3.09	0.61	0.543
Head Length (cm)	17.15 ± 2.72	12.39 ± 3.93	9.38	<0.001
Head breadth (cm)	12.06 ± 1.40	09.43 ± 2.66	7.49	<0.001
Head Circumference (cm)	52.75 ± 1.59	47.05 ± 4.02	11.55	<0.001

As shown in table 2, age, head length, head breadth and head circumference did not differ significantly between male and female patients who have CP (p = 0.694, p = 0.239, p = 0.866, & p = 0.165, respectively). In the comparison group (without CP), mean indices of head size did not differ significantly between males and females (Table 3).

**Table 2:** Sexual Dimorphism in Age, Head Length, Head Breadth, and Head Circumference Among Children with Cerebral Palsy

Variables	Mean ± SD		t-value	*p*-value

	Males (n=40)	Females (n=30)		
Age (year)	4.83 ± 2.98	5.47 ± 3.10	0.87	0.387
Head Length (cm)	11.91 ± 3.56	12.67 ± 3.64	0.88	0.384
Head breadth(cm)	8.98 ± 2.65	10.22 ± 2.65	1.94	0.056
Head Circumference (cm)	46.70 ± 3.80	47.17 ± 4.05	0.49	0.623

**Table 3:** Correlation of Age, Head Length, Head Breadth, and Head Circumference in Children Without Cerebral Palsy

Variables	Mean ± SD		t-value	*p*-value

	Males (n = 40)	Females (n = 30)		
Age (year)	5.43 ± 2.62	5.25 ± 2.13	-0.39	0.694
Head length (cm)	17.49 ± 2.81	16.87 ± 2.63	-1.18	0.239
Head breadth (cm)	12.03 ± 1.54	12.08 ± 1.30	0.17	0.866
Head circumference (cm)	52.51 ± 1.56	52.93 ± 1.61	1.40	0.165

As shown in table 4, among children who had CP, age correlate significantly and weakly with head length (p < 0.0001), head breadth (p < 0.05) and strongly with head circumference (p < 0.0001). Also, the head breadth correlates significantly with head length (p < 0.0001) and head circumference (p < 0.05). However, head circumference correlates weakly with the head breadth (p < 0.05). On the other hand, among children who did not have CP, the age did not correlate significantly with any of the three indices of head size (Table 5). Only the head length correlates moderately with the head breadth (P < 0.001) and weakly head circumference (P < 0.05).

**Table 4:** Correlation of Age, Head Length, Head Breadth, and Head Circumference in Children with Cerebral Palsy

Variables	Age (year)	Head length (cm)	Head breadth (cm)
**Head length (cm)**	0.381*		
**Head breadth (cm)**	0.383**	0.750**	
**Head circumference (cm)**	0.606**	0.261*	0.246*

**Table 5:** Correlation of Age, Head Length, Head Breadth, and Head Circumference in Children with Cerebral Palsy

Variables	Age (year)	Head length (cm)	Head breadth (cm)
**Head length (cm)**	-0.027		
**Head breadth (cm)**	-0.099	0.540**	
**Head circumference (cm)**	0.071	0.235*	-0.009

## Discussions

This study compared the head length, breadth, and circumference of children with cerebral palsy (CP) to those without. Previous research has shown that children with CP generally exhibit poorer growth than their non-CP counterparts^17^-^18^. Poor growth in CP children is primarily attributed to inadequate nutrition due to feeding difficulties^19^-^22^. Children without CP had larger head dimensions than those with CP, as brain damage occurring before or shortly after birth often disrupts brain growth, reducing skull size^23^. Both nutritional and non-nutritional factors have been linked to reduced skull measurements in CP patients, with conditions like plagiocephaly and brachycephaly also affecting skull dimensions in children with CP compared to typical children. Sexual dimorphism refers to the physical differences between males and females. While present in humans, it is less pronounced compared to non-human primates^24^. In this study, no significant differences in head dimensions were found between male and female children with or without CP, consistent with previous findings in a study of Nigerian children aged 5-12 years^25^. Head growth correlates with brain growth^26^. Literature documented poor growth in children with CP growth^27^-^30^. The head dimensions of children with have CP correlate more significantly with age compared to the children without. This brought forth the argument whether CP leads to reduced growth, or if the intrinsic growth potential of children with CP equals that of children with typical development^31^. Positional deformities such as plagiocephaly and brachycephaly which increase the head breadth in the CP relative to normal children explains the correlations observed between head breadths and head lengths in this study.

## Limitations of the Study

The study was unable to classify cerebral palsy into subtypes based on the Gross Motor Function Classification System due to difficulties in accessing CP patients. This led to incomplete patient records and a limited sample size of CP participants. Additionally, while efforts were made to screen control participants based on physical evidence or information provided by parents or guardians, this approach may not always be scientifically reliable.

## Conclusion

This study highlights significant differences in head dimensions between children with cerebral palsy (CP) and those without, with children who did not have CP having larger mean head length, breadth, and circumference. The smaller head size in CP children is linked to brain damage affecting growth, influenced by both nutritional and non-nutritional factors. No sexual dimorphism was found in head measurements. The correlation between head dimensions and age was more significant in CP children, raising questions about their growth potential. Positional deformities, such as plagiocephaly and brachycephaly, may also impact head breadth in children with CP.

## Recommendations

Based on this study's findings, we recommend that Nigerian healthcare providers should closely monitor the growth and nutritional status of children with cerebral palsy (CP) to address potential growth restrictions, especially in head dimensions. Early interventions, including nutritional support and strategies to manage feeding difficulties, should be prioritized to mitigate the impact of both nutritional and non-nutritional factors. Additionally, regular assessments for positional deformities, such as plagiocephaly and brachycephaly, should be implemented to address potential cranial asymmetry. Further research is encouraged to explore the growth potential of children with CP and the role of therapeutic interventions in promoting optimal development.
